# Synergistic Effect of APP and TBC Fire-Retardants on the Physico-Mechanical Properties of Strandboard

**DOI:** 10.3390/ma15020435

**Published:** 2022-01-07

**Authors:** Feiyu Tian, Deliang Xu, Xinwu Xu

**Affiliations:** College of Materials Science and Engineering, Nanjing Forestry University, Nanjing 210037, China; tianfy9758@njfu.edu.cn (F.T.); xudl@njfu.edu.cn (D.X.)

**Keywords:** strandboard, fire retardant, ammonium polyphosphate (APP), 1,3,5-tris(2,3-dibromopropyl)-1,3,5-triazinane-2,4,6-trione (TBC)

## Abstract

This study explored the feasibility of fabricating fire-retardant strandboard with low mechanical properties deterioration to the physico-mechanical properties. A hybrid fire-retardant system of ammonium polyphosphate (APP) and 1,3,5-tris(2,3-dibromopropyl)-1,3,5-triazinane-2,4,6-trione (TBC) was investigated. Thermogravimetric analysis results show that both APP and TBC enhance the thermal stability and incombustibility of wood strands. An infrared spectrum was applied to investigate the effect of flame retardants on the curing behaviors of polymeric diphenylmethane diisocyanate (PMDI) resin. Based on the results of limiting oxygen index (LOI) and Cone calorimetry (CONE), APP and TBC both lead to a higher fire retardancy to strandboard. It is worth mentioning that the two flame retardants lead to evidently differential influences on the modulus of rupture (MOR), modulus of elasticity (MOE), internal bond (IB), and water-soaking thickness swelling (TS) properties of strandboard. Hence, a hybrid flame retardant is prominent in manufacturing strandboard with both good fire retardant and satisfying physico-mechanical properties.

## 1. Introduction

Considering their low-carbon advantages and constructional friendliness, wood constructions are encouraged by Chinese administrations with commercially engineered wood products quantitatively available (e.g., oriented strandboard or OSB [1,2], glued laminated timber or glulam, etc.).

Fire safety tends to be a key factor in wood construction. In 2020, there were 109,000 residential building fires in China, resulting in 1416 death and injuries [3]. The situation seems similarly grim in other parts of the world such as the USA, where timber-framed buildings account for more than 80% of new constructions [4]. In 2018 alone, there were 379,600 residential building fires across the country, resulting in 14,315 death and injuries [5]. Hence, the materials for wood constructions, e.g., OSB, need to be technically treated with fire retardants, such as brominated fire-retardants (BFRs) [6,7,8].

The main fire retarding mechanism of BFRs is decomposition to generate halogen hydride capturing and transfer combustion chain reaction of active radicals (such as ·OH, ·O, ·H) into bromine-free radicals with low activity, resulting in combustion slowing down or termination [9,10]. Meanwhile, some studies further showed that BFRs have positive effects on the mechanical properties of materials treated. Li et al. [11] studied the influence of Brominated polystyrene (BPS) and other flame retardants on Polyamide 6 (PA6). BPS combined with other flame retardants can improve the notch impact strength and tensile strength of PA6. Guo et al. [12] modified rigid polyurethane foams (RPUFs) by brominated benzyl polyols and found that it had little influence on compressive strength. Moreover, the addition of brominated benzyl polyols improves the thermal stability and flame retardancy of RPUFs.

Phosphate flame retardants, typically ammonium polyphosphate, are another family of flame retardants widely used in a variety of materials. These chemicals act mainly depending on the condensed phase retarding mechanism, i.e., the generation of a high-quality thermal insulation char layer, to block oxygen, heat or volatile small particles. However, the pyrolysis of ammonium polyphosphate often affects some mechanical properties of the material. Zhang et al. [13] added phosphorus-containing flame retardant to PU, and the tensile strength increased first and then decreased with the increase of the addition amount. When the addition amount reached 15 wt%, the tensile strength of PU reached the maximum. Xie et al. [14] added phosphate-based flame retardants to PMMA, causing 18.1% deterioration to the tensile strength of PMMA, but having little impact on the tensile modulus and impact strength. Li et al. [15] found, when using poly(P-xylylenediamine spirocyclic pentaerythritol bisphosphonate) (PPXSPB) to modify epoxy resin, the addition of 30 wt% caused 46% reduction to the tensile strength of epoxy resin, but had little effect on the bonding strength. To summarize, two points cause the inverse influence of phosphorous flame retardants on the mechanical properties of the treated materials. Firstly, the agglomeration of flame retardants causes stress concentration. Secondly, the addition of flame retardants affects the pH value of adhesives, leading to the reduction of the bonding strength of adhesives.

In this work, two flame retardants, i.e., APP and TBC, were tried to treat wood strandboard using PMDI resin as the bonding agent. TBC as a hexabromoheterocyclic triazine compound has a high thermal stability, resilience and light degradation resistance [10]. It is similar to PMDI adhesive chemically [8]. Whether the flame retardants affect the curing process of PMDI adhesive was explored. The flame retardant mechanism was preliminarily explained by thermogravimetric analysis and cone calorimetry. Moreover, the physico-mechanical performance of the strandboard was specifically discussed.

## 2. Materials and Methods

### 2.1. Materials

PMDI resin was acquired from Huntsman Co. Ltd. (Shanghai, China). Wood strands were from Baoyuan OSB Co. Ltd. (Hubei, China), which were industrially sliced from small-diameter plantation poplar (*Populus tremula*) logs. The moisture content of wood strands was 8%. Two systems of fire retardants were applied, i.e., ammonium polyphosphate (APP) and tris(2,3-dibromopropyl) isocyanate (TBC) (Yunnan Tianyao chemical industry Co. Ltd., Kunming, China). Acetone (Nanjing Chemical Reagent, Nanjing, China) was invoked as the solvent of the powdered fire retardants, aiming at uniform distribution on wood strands.

### 2.2. Fabrication of Strandboards

**Figure 1:**
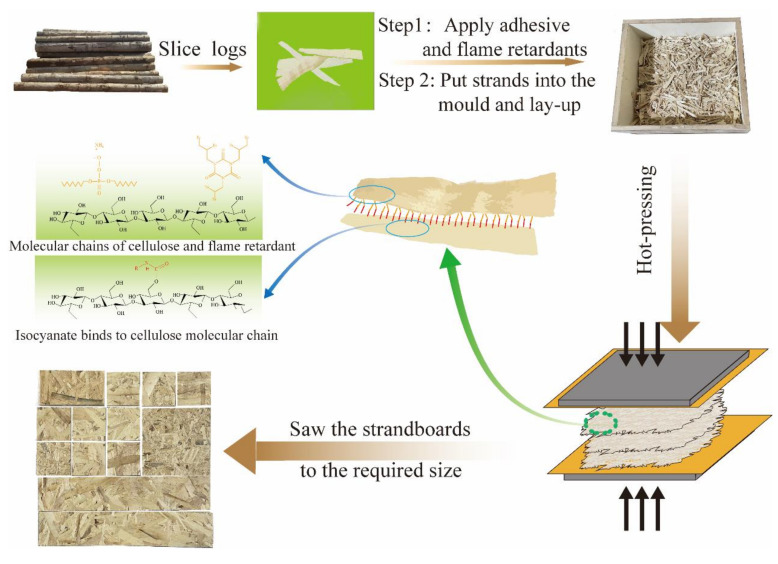
Schematic illustration of strandboards preparation.

The preparation process is shown in Figure 1. Contents of PMDI resin and fire retardants were based on the oven-dry weight of wood strands, followed the regulations in Table 1. The APP, TBC, or hybrid flame retardant was firstly sprayed onto wood strands under stirring for 5 min using an agitator, followed by PMDI adhesive. After lay-up manually, the composite was hot-pressed into a 350 × 350 × 8 mm^3^ board at a target nominal density of 800 kg·m^−3^. Each type of strandboard was prepared for 4 repetitions. The hot-pressing temperature was 180 °C. The whole hot-pressing process lasted for 10 min, 10 MPa for 2 min, 5 MPa for 3 min and 2 MPa for 5 min.

### 2.3. Characterizations

#### 2.3.1. Influence of Flame Retardant(s) on the Curing Behavior of PMDI Resin

A Fourier transform infrared (FTIR) analyzer (VTMR20-010-T, Bruker Corporation, Karlsruhe, Germany) was used to analyze the influence of the flame retardants on the curing process of PMDI resin.

TBC or APP flame retardants (particles through 300 mesh screen), liquid PMDI, and water were mixed in a beaker at a weight ratio of 5:10:2. The beaker was stirred with an electric mixer (JJ-1 60W, Jintan Xicheng Xingri Instrument Factory, Changzhou, China) for 5 min. Then, the beaker containing the sample was removed into an oven at 80 °C. Polymerization reaction of PMDI resin gradually develops, which can be characterized by the change of isocyanate (-NCO) and amino (-NH) groups. Specimens for FTIR analysis were taken from the outermost part of each beaker every 10 min. The total heating time was 50 min until all of the specimens were cured.

To facilitate the analysis of infrared spectral data, the ratio of isocyanates to amino groups is defined as follows:

(1) $$R=\frac{A_{i}}{A_{a}}$$

where *A_i_* is the absorption peak of isocyanate group, *A_a_* is the absorption peak of amino group, and *R* is the ratio.

#### 2.3.2. Distribution of Fire Retardants on Wood Strands

To find out the distribution of fire retardant particles on the wood strands, microscopic scanning was conducted (SEM, Quanta 200 FEI Company, Hillsboro, OR, USA). Wood strands were randomly selected before mat formation and preheated at 60 °C for 3 h. After cooling down to room temperature, the strands were cut into small pieces and fixed on the metal sample table with conductive adhesive. Before observation, the specimens were covered with a thin Aurum film.

#### 2.3.3. Thermogravimetric Analysis

Thermogravimetric Analysis was used to investigate whether flame-retardant would enhance the thermal stability of wood/PMDI and to analyze the flame retardant mechanism of flame retardants. Thermogravimetric (TG) analysis was conducted for the blended sample using the Netzsch STA 409 PC/PG analyzer (Netzsch group, Karlsruhe, Germany) at a heating rate of 10 °C/min and an air flow rate of 30 mL/min ranging from 40 °C to 800 °C.

Wood strands were milled for 5 min into powders (HC-800Y, Wuyi Haina Electric Appliance Co., Ltd., Jinhua, China) and screened out with an 80-mesh screen. The powders were dried at 60 °C for 3 h and cooled at room temperature in a dry dish for 3 h. The content of PMDI was increased and flame retardant was reduced to demonstrate the flame-retardant effect of flame retardants better. Wood powders, liquid PMDI and fire retardant(s) were uniformly mixed at a mass ratio of 100:6:7.

#### 2.3.4. Physico-Mechanical Properties Testing of Strandboards

To investigate the possible influences of fire retardants on the mechanical and physical performance of strandboards, MOE and MOR, IB strength, and 2 h TS were tested in accordance with the Chinese national standard for properties testing methods of wood based-boards, GB/T 17657 [16]. All boards were conditioned at 20 ± 2 °C and 65 ± 5% relative humidity (RH) for one week before cutting specimens for testing. For static bending testing, the specimens of 210 × 50 × 8 mm^3^ were loaded at a middle-length position at a uniform head motion speed of 5 mm/min till fracture. Following that speed, IB strength was acquired through a continuous tensile load perpendicular to the surface of 50 × 50 × 8 mm^3^ specimens. Thickness swelling (TS) testing was conducted for 50 × 50 × 8 mm^3^ specimens, judging the thickness change rate before and after 2 h water soaking at room temperature. 16 repetitions were prepared for IB tests and TS test, while 8 repetitions for MOE and MOR.

#### 2.3.5. Burning Behavior Testing

The burning behavior of various strandboards was tested following LOI method according to the Chinese national standard GB/T 2406.2 (identical to the ISO 4589-2 standard) [17], using an HC900-2 oxygen index meter (Shangyuan Analytical Instrument Co. Ltd., Nanjing, China). Nitrogen and oxygen gases were separately introduced into a burning cylinder (diameter 75 mm, height 400 mm) at a flow rate of 40 mm ± 10 mm/s, and the minimum volumetric percentage of oxygen for igniting and maintaining continuous burning of the specimen is recorded as LOI (%). The specimens were strip-shaped with a dimension of 100 × 7 × 8 mm^3^. Each type of strandboard was prepared for 8 repetitions for LOI tests.

Cone calorimetry (FTTi-Cone 0402) was also used to test the combustion performance of all the strandboards, using the 100 × 100 × 8 mm^3^ specimens according to the ISO 5660-1 standard, in 1800 s at 50 kW/m^2^ heat flux. Each kind of strandboard was prepared 1 sample for CONE tests.

## 3. Results and Discussion

### 3.1. FTIR Analysis

**Figure 2:**
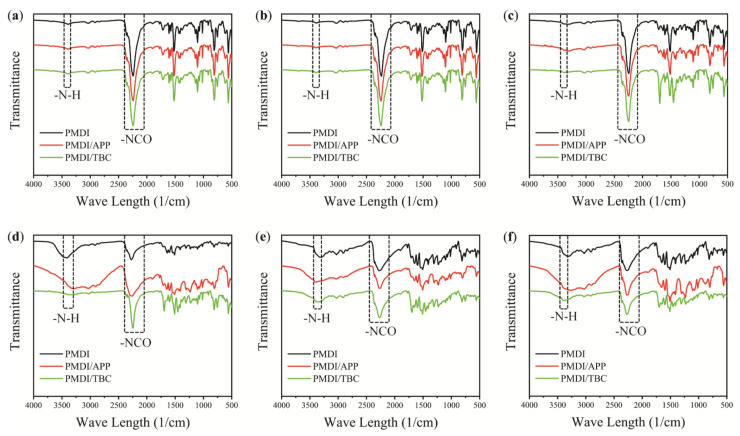
FTIR Spectra of Different Periods (**a**): 0 min (**b**): 10 min (**c**): 20 min (**d**): 30 min (**e**): 40 min (**f**): 50 min.

**Figure 3:**
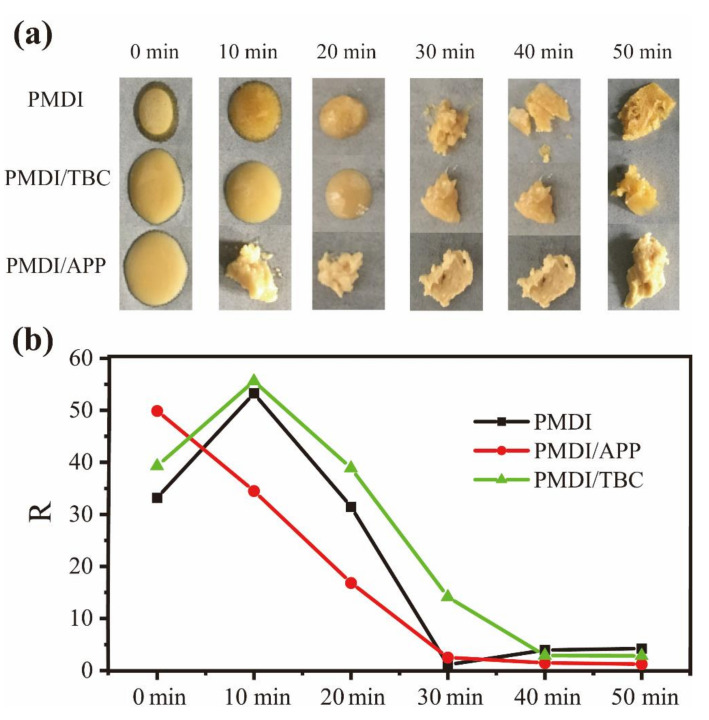
Process Images of (**a**) FTIR Samples Curing Process and (**b**) R Values of Specimens at 80 °C.

ATR-FTIR spectra reveals the changes of chemical groups along with the curing process of PMDI resin. Depending on the polymerization reaction mechanism of isocyanate-water, the absorption peaks of PMDI before and after curing are dominated by -NCO (2270 cm^−1^) and ammonia ester group (3750–3000 cm^−1^), respectively [18]. Combined with Figure 2 and Figure 3, the ratio of isocyanate group to amino group(R) in pure PMDI increases first and then decreases during the whole curing process. In the process of 10~30 min, the amino groups increase rapidly and R value decreases greatly. Macroscopically, it corresponds to higher viscosity. In Figure 2, there is always a peak at 2270 cm^−1^, indicating that the isocyanate group still exists even after PMDI has completely cured. During the curing process, the stretching vibration peak of carbonyl group (1708–1703 cm^−1^) and the bending vibration peak of carbonyl group (1690–1640 cm^−1^) amide II (1540–1530 cm^−1^) in urea increases.

In Figure 3, The R value of PMDI/APP is lower than that of pure PMDI, indicating that the addition of APP increases the viscosity of PMDI at all stages and also leads to the early solidification of PMDI, which is the result the polyphosphoric acid root catalyzed the curing reaction of isocyanate-water.

The spectra of PMDI/TBC and pure PMDI are almost the same, suggesting that there was good compatibility between TBC and PMDI, indicating the addition of TBC has no obvious effect on the curing time of PMDI. But whether there is an influence in the glue bond strength of PMDI still needs to be further investigated.

### 3.2. Distribution of Fire Retardants on Wood Strands

**Figure 4:**
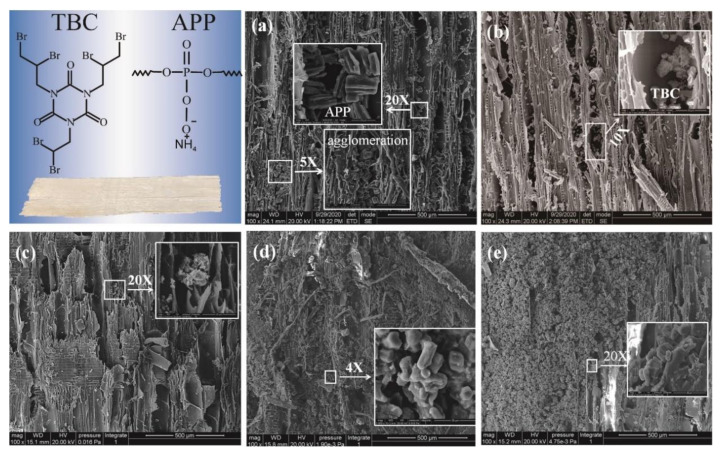
SEM of Fire-Retardant Strands (**a**): FRB/APP (100×, 500× and 2000×) (**b**): FRB/TBC (100× and 1000×) (**c**): CFRB1 (100× and 2000×) (**d**): CFRB2 (100× and 400×) (**e**): CFRB3 (100× and 2000×).

APP particles can penetrate the wood cells, the fracture sites of wood fibers, and the lumen of wood cells. APP particles were widely distributed on the surface of wood, and there was agglomeration among particles (Figure 4a). This may cause the adhesive to fail to hold the wood firmly. According to Image J measurement, the length and width of APP particles are between 23–28 μm and 12–16 μm respectively.

Figure 4b shows TBC is dispersed on the wood surface, mostly in the cell voids, and the penetration is deep in the voids without aggregation. APP agglomeration phenomenon cannot be seen on the surface of wood strands in CFRB1, and the particle size of fire retardant can also be found to be markedly smaller. In CFRB2, the agglomeration phenomenon caused by APP on the surface of wood strands was significantly reduced, but the coverage degree was still high. The combination between APP particles and TBC particles was closer, which made the wood surface flatter at the microscopic level, which was conducive to the later decoration treatment. In CFRB3, agglomeration phenomenon caused by APP was found on the surface of wood strands, but the coverage degree was very high, and APP particles were well distributed in the broken duct channels on the wood surface, and the combination between APP particles and TBC particles was close and the fusion was good.

### 3.3. Physico-Mechanical Properties

The test results showed that the addition of fire retardant had significant interaction with mechanical properties, as shown in Table 2.

After the addition of APP, the deterioration to MOR, MOE and IB of strandboard is clear. Compared to the control group, MOR and IB strength in the FRB/APP group were reduced by 39.49% and 27.35%, respectively. Agglomeration occurs on the surface of wood strands treated by APP causing the concentration of stress in strandboards and deterioration of MOR. This can be seen in by FTIR analysis. The addition of APP makes PMDI solidify in advance. When it happens in the hot-pressing process, the surface part of strandboard will cure before the core layer, which will decrease the bonding performance and lead to the deterioration of mechanical properties. However, the FRB/APP group showed a better thickness swelling rate than the control groups. This is due to the high degree of polymerization (>1000) of APP, which causes poor water solubility and shows a hydrophobic effect.

TBC reduces the MOR of strandboard but increases the IB strength and MOE. Compared to the control group, the MOR of FRB/TBC strandboard was decreased by 29.13%.

Due to the rigid triazine ring, TBC increased the MOE of strandboards. TBC is a hydrophobic organic flame retardant, which is the main reason for the small value of TS. When APP and TBC are simultaneously added into strandboards, it was found that the more TBC added, the better MOE is.

Compared to the control group, MOR of CFRB1 was reduced by 31.29%, CFRB2 by 35.95%, and CFRB3 by 34.19%. In the terms of MOE, TBC still works after blending. The MOE of CFRBs shows a decreasing trend when the content of TBC decreases. However, IB strength and TS of strandboards are not as obvious as MOE. Compared to the control group, the internal bond strength of CFRB1 was decreased by 29.06%, CFRB2 by 20.51%, and CFRB3 decreased by 23.93%. Which is the result of APP agglomeration between the glue lines. Among the hybrid strandboard, CFRB1 shows the best physico-mechanical properties.

### 3.4. Thermal Stability of Specimens

**Figure 5:**
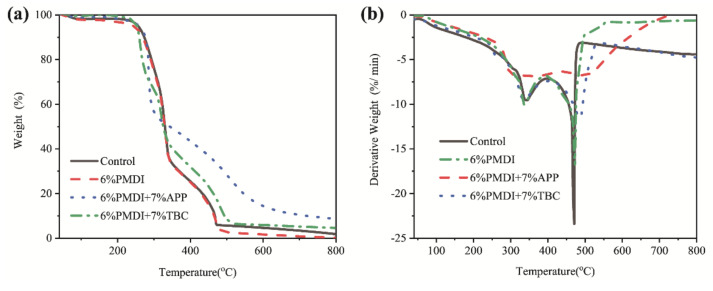
Thermogravimetric Curve (**a**) and Derivative Thermogravimetric Curve (**b**) of Fire-retardant Strandboards.

The effect of PMDI on the thermal stability of wood powder was tested by TGA. Wood powders were the main component in all specimens, so the pyrolysis curves show a similar trend to that of the Control group (Figure 5). The first stage (60–220 °C) is due to the loss of small molecules such as thermally unstable branched chains on water and hemicellulose. The second stage (220–340 °C) is the main pyrolysis stage. Cellulose and hemicellulose produce a large number of low molecular compounds at high temperatures, along with char. The third stage (340–470 °C) comes from the continuous decomposition of cellulose, hemicellulose, lignin and other main components and char production. After 470 °C, most of the charred portion burns steadily [19]. The addition of 6%PMDI resulted in lower combustion residues, suggesting that PMDI had a side effect on char layer quality.

The addition of APP significantly improved the thermal stability of the wood. TBC also has a positive effect on the thermal stability of wood. Table 3 compares the characteristic temperature and residual char content of different materials. The reason why it takes higher temperature for APP to initial degradation temperature (T_5%_) of APP is that the NH_3_ and polyphosphate acid generated from the endothermic decomposition reaction of APP in the first stage of the pyrolysis process (60–220 °C). NH_3_ covers part of the wood surface and isolating air. In the next weight-loss stage (220–310 °C), due to the dehydration of hemicellulose and cellulose polyphosphate acid and the promotion of char production, the pyrolysis temperature was higher and the pyrolysis rate was effectively reduced. The flame retardant effect of TBC is not so obvious as that of APP. According to the increase of char yield after the addition of TBC, it can be judged preliminarily that TBC has a condense-phase flame retardant mechanism.

In general, the addition of PMDI promoted the thermal decomposition of wood, while both flame retardants improved the thermal stability of wood. APP is better than TBC, especially in the term of char yield.

### 3.5. Limited Oxygen Index (LOI)

LOI is a convenient tool to assess the flammability of materials. the LOI of strandboards treated with flame retardants was all higher than 27% (Table 4). The limited oxygen index of FRB/APP is higher than FRB/TBC. Among the hybrid flame retardant stranboards, it shows a similar pattern. The more APP contains, the higher LOI value is.

The role of APP in the fire combustion of strandboard is divided into two steps. Firstly, APP releases ammonia and generates polyphosphate acid, which would promote wood cellulose and hemicellulose dehydration, absorb heat and accelerate the charring process. Secondly, APP loses water and forms an expansion layer covering the wood surface to isolate heat and air. In CFRBs, the quality of char is better and less flammable gas flees from wood when it contains more APP. This is the reason why CFRB1 shows the smallest value in LOI test. The main effect of adding TBC into strandboard during combustion is to capture high-energy free radicals and generate non-flammable gas. It has a very limited effect in accelerating the charring processing.

### 3.6. Cone Calorimetric Analysis

**Figure 6:**
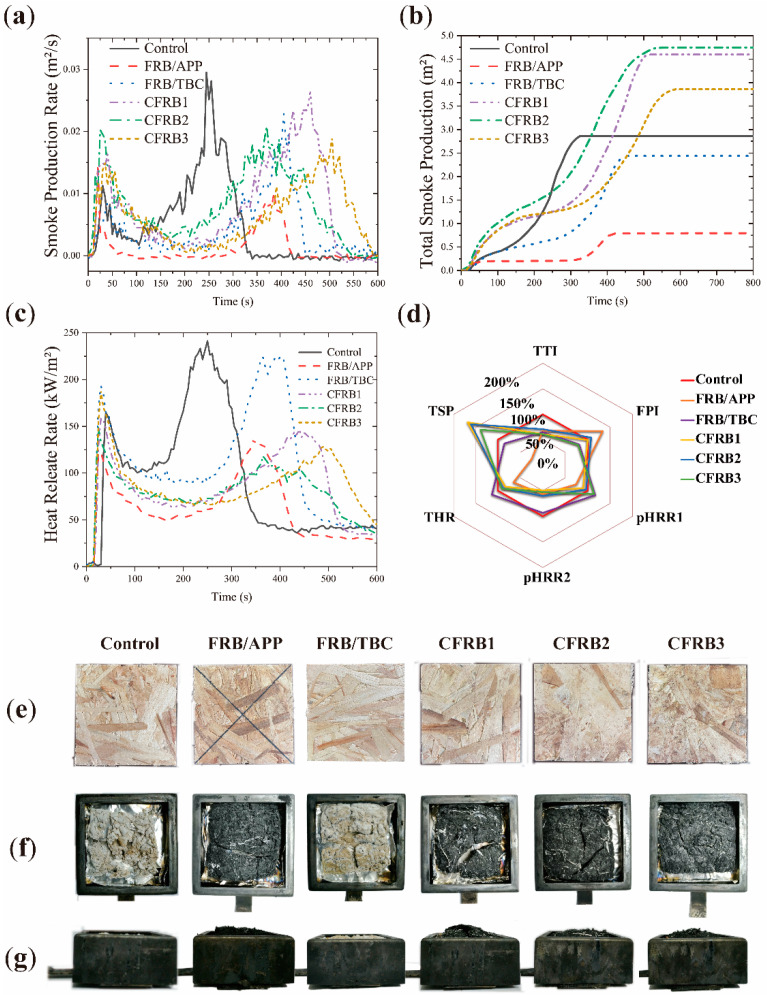
Flame retardancy of the wood strandboards with and without flame retardants. (**a**–**c**) Smoke Production Rate (SPR), total Smoke Production, Heat Release Rate (HRR) during the Cone Calorimetric Test. (**d**) The comprehensive combustion performance of flame retardant specimens compared with control group. (**e**–**g**) Images of CONE test samples before and after combustion, top views of CONE test samples before combustion; top and side views of char residues.

The combustion performance of strandboards was comprehensively analyzed according to Table 5 and Figure 6. As can be seen from Figure 6d, the ignition time (TTI) of strandboards enhanced by flame retardants is shorter than that of the control group, because flame retardants catalyze thermal decomposition, reduce thermal decomposition temperature, and release small molecule volatile substances in advance [20].

The heat release rate (HRR) of strandboards can be divided into two peaks (Figure 6c). The formation of the first peak is due to the continuous heating and combustion of the strandboard surface, which gradually formed the thermal insulation char layer. As a result, HRR is greatly reduced. Only after the char layer was destroyed by high temperature did the inner of the strandboards begin to release heat and form a second peak. As can be seen in Figure 6 the addition of APP reduced both pHRR1 and pHRR2. This is because the phosphoric acid generated by the thermal decomposition of phosphorus groups acts as a nucleophilic center to promote the cyclization of char layer. After phosphoric acid was coated on the char layer, the oxidizable active center on the char layer was passivated and smoldering behavior was inhibited.

The addition of TBC prolongs the arrival of pHRR2, as TBC releases non-flammable gases during the combustion decomposition process to play the role of free radical collector, making strandboard combustion intensity inferior to the control group.

Among the compound groups, the arrival time of CFRB3 was the last, and the pHRR2 of the three compound combinations was at a similar level. This indicates that APP can also release free radical trapping gas when the char layer is broken, but the flame retardancy effect of free radical trapping is not particularly outstanding compared with the flame retardancy effect of condensed phase mechanism for high-quality char layer being formed.

Smoke release rate and total smoke production are both important indexes of combustion performance. The peak time of SPR and HRR was consistent. They are all related to the insulating char layer. When the char layer is dense, there are fewer emission channels in strandboard. When the char layer is broken, the accumulated flue gas is released in large quantities. The peak SPR of the control group was 0.029 m^2^/s, TSP was 2.8 m^2^, TBC and APP were 0.0228 m^2^/s and 0.0147 m^2^/s, respectively. The maximum SPR in the compound groups is 0.0265 m^2^/s from CFRB2. CFRB3 was 0.0189 m^2^/s, which was the lowest SPR in the composite group. Compared with the control group, SPR and TSP in TBC group decreased 21.38% and 14.29%, respectively, and SPR and TSP in APP group decreased 50.09% and 75%, respectively. However, the SPR of composite flame retardant did not decrease obviously, and TSP increased to different degrees. The TSP high indicated that it was not easy to produce visible fire in the combustion process.

Fire Performance Index (FPI) is the proportion of TTI and the maxima of the initial peak intensity of the heat release rate (HRR) curve. The larger the FPI, the better the flame retardant effect [21]. Combining the results of HRR, it can be concluded that FRB/APP has the best flame retardant effect in all combinations, and CFRB2 has the best flame retardant effect in composite combinations.

## 4. Conclusions

PMDI-bonded strandboard with APP and/or TBC retardants were studied. Addition of the retardants enhances the thermal stability and incombustibility of wood strands and affects the curing behavior of PMDI resin as well. A valuable finding is that APP and TBC lead to evidently differential influences on the bending and internal bond properties of strandboard. FTIR indicated that the addition of APP would make PMDI curing in advance, and the macroscopic mechanical performance was the decrease of IB strength. However, the addition of TBC has little influence on PMDI curing process, and the mechanical properties of FRB/TBC were better than FRB/APP, especially in IB strength. In hybrid groups, CFRB2 shows the best physico-mechanical properties. Its MOR was 30.54 MPa, MOE was 4158.6 MPa, IB was 0.93 MPa. In burning behavior tests, both LOI and CONE results showed that the more APP contained, the better flame retardant effect. CONE also indicated that the combination of APP and TBC could not only improve the quality of char layer, but also enhance the flame retardant effect from the perspective of gas phase mechanism. Combined with mechanical and combustion test results, CFRB2 is the optimal combination scheme. A combination scheme of APP and TBC shows its prominent future in manufacturing fire-retardant strandboard with good comprehensive performance.

## Figures and Tables

**Table 1:** Synthesis condition of fire-retardant strandboards.

Specimens	PMDI, wt%	Fire Retardants, Phr	
		APP	TBC
Control	4		
FRB/APP	4	10	
FRB/TBC	4		10
CFRB1	4	2.5	7.5
CFRB2	4	5	5
CFRB3	4	7.5	2.5

**Table 2:** Mechanical and physical properties of fire-retardant strandboard.

Specimens	Modulus of Rupture (MPa)	Modulus of Elasticity (MPa)	Internal Bond Strength (MPa)	Thickness Swelling (%)
Control	47.68 (4.65) *	4482.68 (426.07)	1.17 (0.34)	5.41 (0.03)
FRB/APP	28.85 (4.42)	3938.42 (740.39)	0.85 (0.08)	4.19 (0.01)
FRB/TBC	33.79 (3.11)	5130.94 (643.10)	1.18 (0.27)	1.41 (0.01)
CFRB1	32.76 (3.66)	4232.16 (823.31)	0.87 (0.21)	3.93 (0.02)
CFRB2	30.54 (8.48)	4158.60 (774.18)	0.93 (0.22)	3.83 (0.01)
CFRB3	31.38 (7.14)	4065.96 (829.51)	0.89 (0.12)	4.04 (0.01)

* Values in parentheses are standard deviation for 8 repetitions for MOR and MOE, 15 repetitions for IB strength and TS.

**Table 3:** Thermogravimetric analysis results.

Specimens	T_5%_ (°C)	T_peak1_ (°C)	T_peak2_ (°C)	Char at 800 °C (wt%)
Control	254.58	343.19	470.61	1.91
6%PMDI	242.26	335.67	472.98	0.19
6%PMDI + 7%APP	257.72	348.06	492.03	8.67
6%PMDI + 7%TBC	252.56	333.50	484.76	4.64

T_5%_: decomposition temperature at 5% mass loss; T_peak1_: the first peak temperature; T_peak2_: the second peak temperature.

**Table 4:** LOI of Different Strandboards.

Specimens	Limited Oxygen Index (%)
Control	25.19 (0.29) *
FRB/APP	34.23 (0.22)
FRB/TBC	29.10 (0.30)
CFRB1	30.11 (0.11)
CFRB2	30.97 (0.27)
CFRB3	33.77 (0.21)

* Figures in parentheses are standard deviation values for 8 repetitions.

**Table 5:** Cone calorimetry test results of strandboards.

Scheme	TTI (s)	FPI (m^2^s/kW)	pHRR1 (kW/m^2^)	pHRR2 (kW/m^2^)	THR (MJ/m^2^)	TSP (m^2^)
Control	31	0.19	165.29	241.37	56.84	2.8
FRB/APP	21	0.17	122.88	136.86	37.76	0.7
FRB/TBC	20	0.10	193.13	225.7	64.83	2.4
CFRB1	19	0.13	148.58	117.85	47.94	4.7
CFRB2	22	0.14	159.12	146.77	50.84	4.5
CFRB3	19	0.10	188.01	128.16	52.14	3.9

TTI: time to ignition; FPI: fire performance index; pHRR1: the initial peak of heat release rate; pHRR2: the second peak of heat release rate; THR: total heat release; TSP: total smoke production.

## Data Availability

Not applicable.
